# Potential of siRNA-Bearing Subtilosomes in the Treatment of Diethylnitrosamine-Induced Hepatocellular Carcinoma

**DOI:** 10.3390/molecules28052191

**Published:** 2023-02-27

**Authors:** Fauzia Jamal, Ghufran Ahmed, Mohammad Farazuddin, Ishrat Altaf, Saba Farheen, Qamar Zia, Asim Azhar, Hira Ahmad, Aijaz Ahmed Khan, Satyanarayana Somavarapu, Anshu Agrawal, Mohammad Owais

**Affiliations:** 1 Interdisciplinary Biotechnology Unit (IBU), Aligarh Muslim University, Aligarh 202002, India; 2 Division of Microbiology, ICMR-Rajendra Memorial Research Institute of Medical Sciences, Patna 800007, India; 3 Mary H. Weiser Food Allergy Center, University of Michigan, Ann Arbor, MI 48109-1316, USA; 4 Health and Basic Science Research Centre, Majmaah University, Majmaah 11952, Saudi Arabia; 5 Neat Meatt Biotech Private Limited, Bio-NEST-UDSC, University of Delhi (South Campus), New Delhi 110021, India; 6 Department of Zoology, Aligarh Muslim University, Aligarh 202002, India; 7 Department of Anatomy, Jawaharlal Nehru Medical College, Aligarh Muslim University, Aligarh 202002, India; 8 UCL School of Pharmacy, University College London, 29-39 Brunswick Square, London WC1N 1AX, UK; 9 Division of Basic and Clinical Immunology, Department of Medicine, University of California Irvine, Irvine, CA 92697, USA

**Keywords:** siRNA, COX-2, subtilosome, hepatocellular carcinoma, apoptosis

## Abstract

Therapeutics, based on small interfering RNA (siRNA), have demonstrated tremendous potential for treating cancer. However, issues such as non-specific targeting, premature degradation, and the intrinsic toxicity of the siRNA, have to be solved before they are ready for use in translational medicines. To address these challenges, nanotechnology-based tools might help to shield siRNA and ensure its specific delivery to the target site. Besides playing a crucial role in prostaglandin synthesis, the cyclo-oxygenase-2 (COX-2) enzyme has been reported to mediate carcinogenesis in various types of cancer, including hepatocellular carcinoma (HCC). We encapsulated COX-2-specific siRNA in *Bacillus subtilis* membrane lipid-based liposomes (subtilosomes) and evaluated their potential in the treatment of diethylnitrosamine (DEN)-induced hepatocellular carcinoma. Our findings suggested that the subtilosome-based formulation was stable, releasing COX-2 siRNA in a sustained manner, and has the potential to abruptly release encapsulated material at acidic pH. The fusogenic property of subtilosomes was revealed by FRET, fluorescence dequenching, content-mixing assay, etc. The subtilosome-based siRNA formulation was successful in inhibiting TNF-α expression in the experimental animals. The apoptosis study indicated that the subtilosomized siRNA inhibits DEN-induced carcinogenesis more effectively than free siRNA. The as-developed formulation also suppressed COX-2 expression, which in turn up-regulated the expression of wild-type p53 and Bax on one hand and down-regulated Bcl-2 expression on the other. The survival data established the increased efficacy of subtilosome-encapsulated COX-2 siRNA against hepatocellular carcinoma.

## 1. Introduction

Hepatocellular carcinoma (HCC) is considered the fifth most common malignancy worldwide [1,2] and shows a multi-fold increase in its occurrence. More importantly, it is anticipated to remain one of the primary carcinomas affecting the human population in the next two decades [3]. The major risk factors for HCC include chronic infection due to hepatitis viruses (B and C clades), alcohol-induced cirrhosis, genetic hemochromatosis, non-alcoholic steato-hepatitis, and exposure to potent hepato-carcinogens, such as aflatoxin B1, etc. [4].

Cyclo-oxygenase-2 (COX-2) is an important enzyme that seemingly plays a role in cancer development. The enzyme has been reported to regulate many processes, such as angiogenesis, apoptosis, inflammation, immuno-suppression, and invasiveness [5,6]. Earlier studies suggested an up-regulated expression of COX-2 in various human cancers involving the liver, colon, gastric, esophagus, pancreas, and breast tissues [7]; by contrast, it remains undetectable in normal healthy tissues. Furthermore, the over-expression of COX-2 has been linked to the induction of tumorigenesis in animal models. Studies have suggested that cancer cells with elevated levels of COX-2 become unresponsive to apoptotic stimuli [8,9]. A significant body of literature has supported the role of this enzyme in sustaining cell homeostasis in healthy versus cancer cells [7,9,10]. In brief, the COX-2-prostanoid pathway causes inflammation and is responsible for liver diseases, such as cirrhosis and HCC [11,12]. Considering these factors, we chose COX-2 as a target with the assumption that its siRNA-mediated silencing may serve as a measure to inhibit diethylnitrosamine (DEN)-induced HCC in model animals.

Despite showing great potential in suppressing oncogenes in in vitro and in vivo systems, RNAi technology has encountered many obstacles when translated into pre-clinical and clinical settings [13,14,15]. The highly poly-anionic nature and the bulky size of unmodified siRNA molecules restrict their penetration through hydrophobic plasma membranes, resulting in reduced intracellular delivery [16,17]. The siRNA is also prone to self-degradation, has a very short half-life, and is easily degraded in the systemic circulation, thereby exhibiting rapid clearance from systemic circulation [18]. Moreover, unmodified naked siRNA molecules also exhibit remarkable systemic toxicity [19]. In addition, some of the sequence motifs in siRNA molecules can induce the production of type I interferon and proinflammatory cytokines upon their interaction with specific immune cells [20].

Various synthetic and natural nanoparticle-based delivery systems have demonstrated distinct prospects for the safe and efficient delivery of synthetic siRNAs. Interestingly, siRNA-based novel carriers manifest variable efficacy and safety profiles. Among various nanoparticle-based formulations, liposomes have proven effective in delivering siRNA into tumor tissues by improving the stability and bioavailability of the encapsulated material [21,22]. In our earlier studies, we reported that lipid vesicles derived from bacterial lipids have the potential to deliver encapsulated materials to the cytosol of the target cells upon interaction due to strong membrane–membrane fusion [23,24,25]. We also used in-house-developed lipid vesicles as a drug- and vaccine-delivery platform against various infectious diseases and cancer [24,25,26,27].

Considering the fusogenic property of lipids derived from lower organisms, in the present study, we developed a delivery vehicle employing *B*. *subtilis*-plasma-membrane lipids that might efficiently deliver entrapped COX-2 siRNA to the cytosol of the target cells [28]. In our pilot studies, we observed that *B. subtilis*-lipid-derived nano-particles (subtilosomes) are biocompatible, biodegradable, and devoid of any host-immune-system-related complexity. Herein, we examine the efficacy of COX-2 siRNA-loaded subtilosomes in the treatment of DEN-induced HCC in model animals. The higher efficacy of the subtilosome-based novel-drug-delivery vehicle, derived from *Bacillus subtilis* (*B*. *subtilis*) lipids, can be justified on the premise that the as-formed lipid vesicles mimic nano cells.

## 2. Results

### 2.1. Characterization of Subtilosomes-siRNA Based Nanoparticles

**Figure 1:**
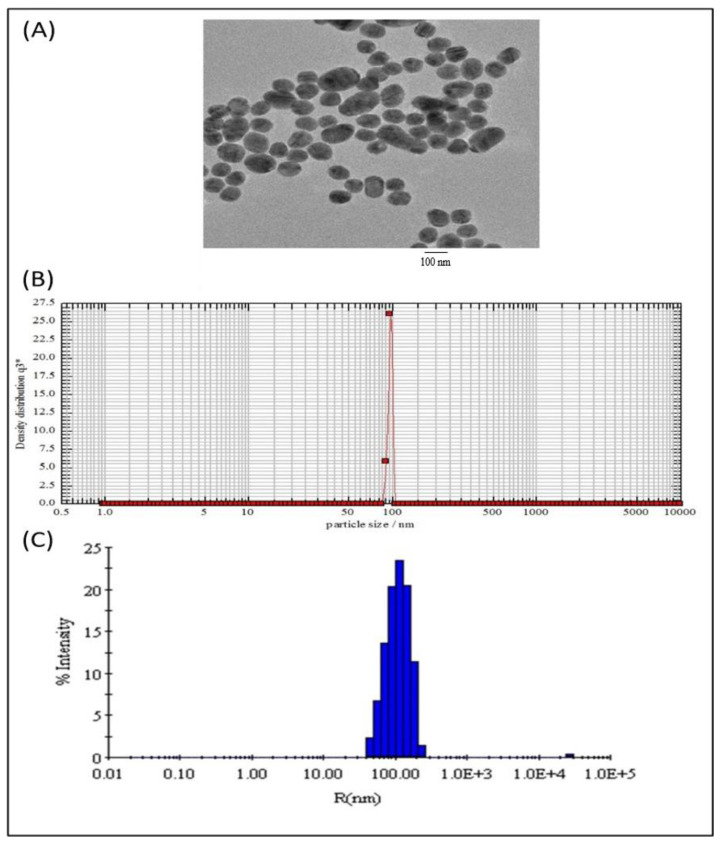
Size distribution and morphology of subtilosome-based siRNA nanoparticles. (**A**) Transmission electron microscopy showed that the siRNA-subtilosomes were spherical in shape, with an average size of 95 ± 10 nm. (**B**) Particle-size distribution of siRNA-subtilosomes assessed by photon-correlation spectroscopy. (**C**) The Z-average diameter of the siRNA-subtilosomes determined by dynamic light scattering measurements was 105 ± 0.7 nm.

The *B*. *subtilis*-membrane lipids were isolated following a method described elsewhere with slight modifications [29,30]. The subtilosomes constructed from the *B*. *subtilis* total lipids had an entrapment efficiency of 27 ± 4.2% for COX-2 siRNA. By contrast, the entrapment efficiency of the COX-2 siRNA in the PC-liposomes was 23 ± 2.0%. In addition, the negative zeta potential (−30.67 ± 0.4) of the as-formed lipid vesicles suggested that a repulsive force (entropic) prevented the agglomeration of the as-formed subtilosome. The Z-average diameter of the as-synthesized siRNA subtilosomes, as determined by dynamic light scattering measurements, was 105 ± 0.7 nm. However, electron microscopy showed that the as-synthesized siRNA subtilosomes had spherical shapes, with an average size of 95 ± 10 nm (Figure 1).

### 2.2. Release Kinetics of siRNA from As-Synthesized Nanoparticles

**Figure 2:**
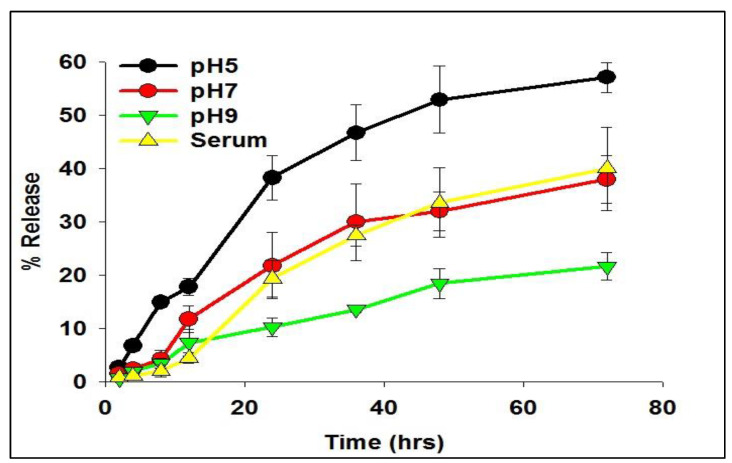
Time course of siRNA release from subtilosomes for extended period. The siRNA-loaded subtilosomes were incubated in sterile 20-millimolar phosphate-buffered saline of various pH (pH 5, 7, and 9) for an extended period. The co-incubation of siRNA-loaded subtilosome with serum components showed release kinetics of the encapsulated siRNA, similar to that of plain (no serum components) subtilosome. The released siRNA in the surrounding medium was quantified over time by HPLC, as described in the Section 4. Each time point represents the mean of triplicate readings ± standard deviation.

The release kinetics of the siRNA-entrapped subtilosomes exhibited a slow and sustained release profile over an extended period. The in-house-prepared subtilosomes maintained their stability and integrity at neutral or alkaline pH change for a time period of more than 50 h (Figure 2). In the first 10 h, approximately 10–20% of the total loaded siRNA was released at pH 7 and 9. Approximately 21 and 38% of the siRNA was released after 72 h from the subtilosome-based formulation incubated at pH 9 or 7, respectively, whereas the incubation at pH 5 resulted in the release of ~57% of siRNA. The subtilosome formulation remained stable in the presence of plasma components for a period of more than 70 h, with a release of less than 40% of the siRNA (Figure 2).

### 2.3. Analysis of Fusogenic Property of Subtilosome

**Figure 3:**
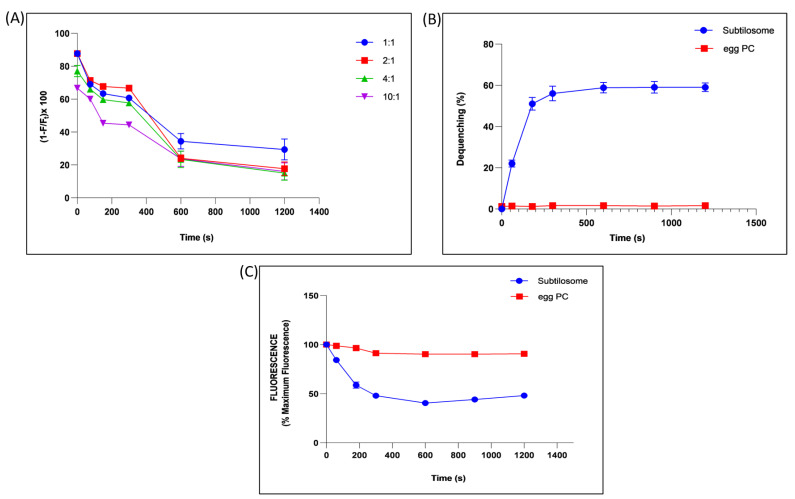
Fusogenic property of subtilosome. (**A**) Time-dependent effect on the efficiency of FRET between NBD and rhodamine grafted on the subtilosome surface upon mixing the labelled subtilosomes with increasing amounts of unlabelled subtilosomes (labelled-to-unlabelled ratios were taken as 1:1, 1:2, 1:4, 1:10). (**B**) Interaction of Rh-PE-labelled subtilosomes or egg-PC liposomes with their unlabelled counterparts. Subtilosomes or egg-PC liposomes containing 5 mol% Rh-PE were allowed to interact with unlabelled form of the same types of the vesicles in a ratio of 1:10. The fluorescence associated with the labelled subtilosomes or egg-PC liposomes was monitored for up to 20 min using excitation and emission wavelengths of 536 and 585 nm, respectively. (**C**) Time-dependent quenching of the ANTS fluorescence by mixing the ANTS-containing subtilosomes or egg-PC liposomes with DPX-containing subtilosomes or egg-PC liposomes in a ratio of 1:10. The excitation and emission wavelengths used were 380 and 540 nm, respectively. Values are the means of three independent experiments ± S.D.

The potential of the subtilosomes to induce membrane–membrane fusion was assessed by monitoring the mixing of the lipid vesicles. The fusion efficiency was determined by measuring efficiency of FRET between NBD (photon donor, absorption at 470 nm, emission at 520 nm) and rhodamine (photon acceptor, absorption at 540 nm, emission at 585 nm). These probes were grafted on liposome surfaces by including NBD-PE and Rh-PE in *B. subtilis* lipids. The subtilosomes containing these two fluorophores (labelled) were diluted with increasing amounts of subtilosomes free of these fluorophores (unlabelled). The efficiency of FRET was calculated by measuring the NBD fluorescence, as described in Materials and Methods. As the efficiency of FRET between these fluorophores was shown to depend on their concentration (*cf.* spatial vicinity) in the vesicle bilayer, it was expected that any fusion between the labelled and unlabelled subtilosomes would lead to dilution of the probes and, consequently, to a reduction in the FRET efficiency. The efficiency of FRET of subtilosomes decreased with an increase in the incubation time, as well as with the ratio of the labeled to unlabeled subtilosomes. We observed that the efficiency decreased to ≈20% when the labelled/unlabelled ratio was 1:10 at 20 min (Figure 3A).

Vesicle–vesicle fusion was also studied by including a self-quenching concentration (5 mol%) of Rh–PE in subtilosomes or egg-PC liposomes. Fusion of the labelled subtilosomes or egg-PC liposomes with overwhelming numbers of unprobed counterparts resulted in a significant ≈ 60% dequenching of the fluorescence in subtilosomes, while no significant dequenching was found in the fluorescence of egg-PC liposome (Figure 3B).

The membrane-fusion potential of subtilosome was further validated by monitoring the mixing of the aqueous contents during fusion by measuring quenching of the ANTS fluorescence by the water-soluble quencher DPX. Incubation of the ANTS containing subtilosome with a 10-fold excess of DPX-containing subtilosome resulted in ≈50% quenching of the ANTS fluorescence in 20 min (Figure 3C). However, no such quenching of the ANTS fluorescence was observed when ANTS-containing egg-PC liposomes were incubated with a 10-fold excess of the DPX-containing egg-PC liposomes under identical conditions.

### 2.4. COX-2-siRNA-Bearing Subtilosomes Mediated Liver-Enzyme Depletion in the Experimental Animals

**Figure 4:**
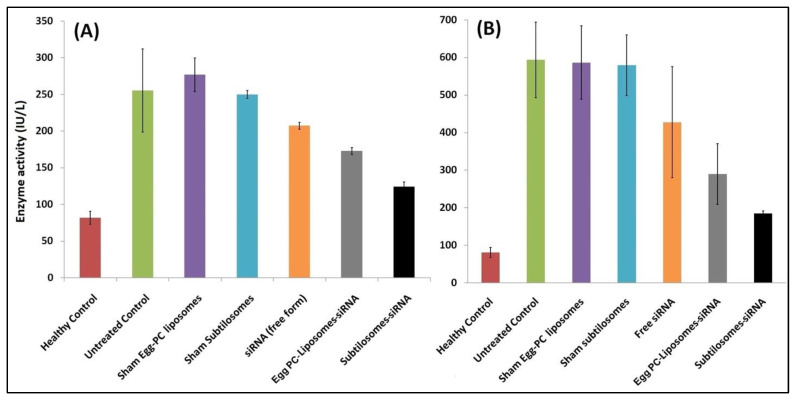
Liver-enzyme levels in animals treated with different siRNA-bearing liposomal formulations. Enzyme activities were measured as described in Materials and Methods. Data represented here are the means of three separate experiments ± SD. (**A**) ALT (subtilosome siRNA versus free siRNA *p* < 0.05, PC-liposome siRNA versus free siRNA *p* value not significant, subtilosome siRNA versus PC-liposome siRNA *p* < 0.05), (**B**) AST (subtilosome siRNA versus free siRNA *p* < 0.005, PC-liposome siRNA versus free siRNA *p* < 0.05, subtilosome siRNA versus PC-liposome siRNA *p* < 0.005).

The COX-2 has been reported to mediate the proliferation process in liver-cancer cells. This adversely affect the expression and functioning of the liver enzymes. We examined the activity of ALT and AST, the two crucial enzymes associated with liver function, in the sera of animals treated with various siRNA-based formulations. The liver-cancer cells expressed a copious amount of the enzymes, as evidenced by the levels of two enzymes in the control group of DEN-exposed animals (not treated with siRNA-based formulations). We found that the subtilosomes mediated the delivery of COX-2 specific siRNA helped in the down-regulation of the expression and activities of the enzymes (*p* value < 0.01). The PC-liposome-entrapped siRNA also helped in the enzyme regression; however, its efficacy was lower than that of the subtilosomes (*p* value < 0.05). The naked siRNA also demonstrated a reduction in enzyme activity, although in a statistically non-significant manner (Figure 4).

### 2.5. The siRNA-Bearing Subtilosomes Induce Apoptosis in Hepatocellular Carcinoma Cells

**Figure 5:**
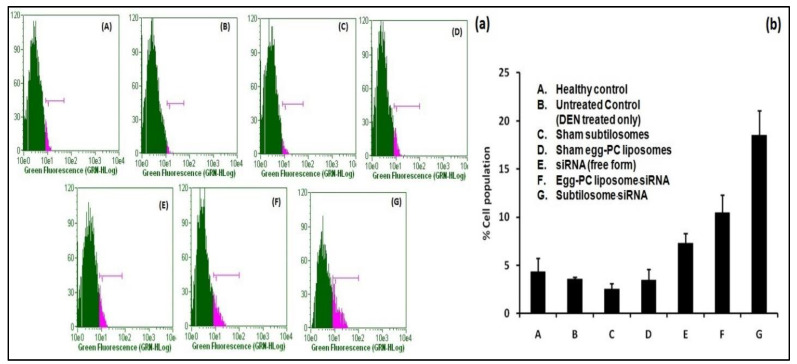
Apoptosis in liver cells analyzed by BrdU staining. Staining was performed using a BrdU staining kit (BD Biosciences), as described in the Section 4. (**a**) Stained-cell population from various groups of experimental animals treated with siRNA formulations where (**A**–**G**) represent healthy control, untreated control (DEN treated only), sham subtilosome treated, sham egg-PC liposome treated, free siRNA treated, egg-PC liposome siRNA treated and subtilosome siRNA treated groups, respectively. (**b**) The total percentage cell population (depicted as bar diagram) observed in various experimental groups.

The animals afflicted with HCC were treated with various siRNA-based formulations. Among the different siRNA-based formulations tested, the subtilosome-based siRNA formulation was the most effective in delivering encapsulated siRNA at the target site and induced the maximum apoptosis (18.53%) of the cancer cells, followed by the egg-PC liposomes (10.48%). The naked siRNA showed only a mild effect on apoptosis induction (7.53%), while the sham subtilosomes and sham egg-PC liposomes failed to induce apoptosis of cancer cells (Figure 5).

### 2.6. Effect of siRNA-Bearing Subtilosomes on the Expression of TNF-α

**Figure 6:**
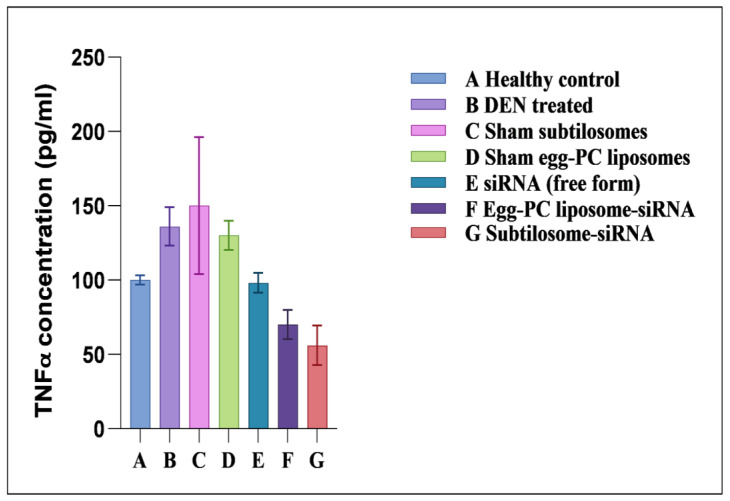
TNF-α-expression profiles of animals treated with different siRNA formulations. TNF-α levels were measured in plasma, as described in Materials and Methods. Data represented here are the mean of three separate experiments ±SD (siRNA subtilosomes versus free siRNA *p* < 0.05, siRNA-PC liposomes versus free siRNA *p* < 0.05, siRNA subtilosomes versus siRNA-PC liposomes *p* value not significant).

The COX-2 was reported to regulate TNF-α expression in the host. The subtilosome-encapsulated siRNA significantly inhibited TNF-α expression, whereas the free form of siRNA produced a very mild effect (Figure 6). The control groups, such as the sham PC liposomes and sham subtilosomes, exhibited no effect on TNF-α expression (data not shown).

### 2.7. Expression of Pro/Anti-Apoptotic Factors

**Figure 7:**
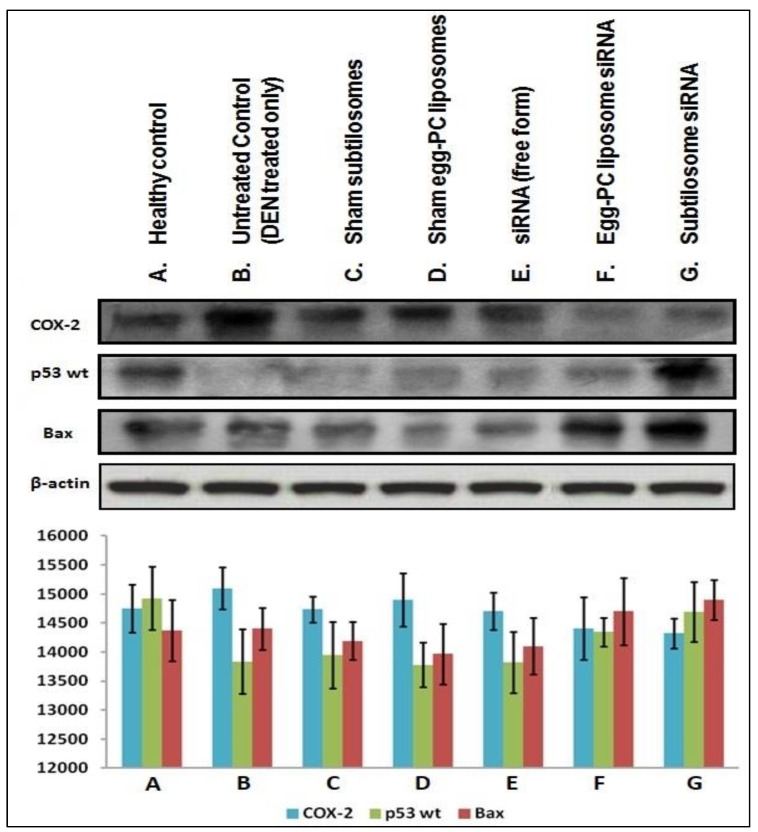
Expression profiles of COX-2 and various apoptotic molecules in the liver cells isolated from various si-RNA formulation-treated animal groups. Western-blot assay of COX-2, p53 WT, and Bax in a liver extract from mice treated with multiple COX-2-siRNA-bearing liposomal formulations.

As shown in Figure 7, the subtilosome-based siRNA formulation inhibited the expression of COX-2 in the cancer cells. The egg-PC-liposome-based siRNA formulation was also successful in the containment of the COX-2 enzyme in the cancer cells, although less efficiently than the subtilosome-based siRNA formulation (subtilosome siRNA vs. egg-PC-liposome siRNA; *p* value < 0.05). While the treatment with naked siRNA inhibited the expression of COX-2 non-significantly (subtilosome siRNA vs. naked siRNA; *p* value < 0.01), the sham subtilosome-based formulation and the untreated control groups failed to inhibit COX-2 expression at the same dose. The down-regulation of the COX-2 expression was most prominent in the case of the subtilosome-siRNA nanoparticles, followed by that of the egg-PC-liposomal siRNA formulation. The potential of COX-2-mediated apoptosis was further established by determining the expression pattern of p53wt and Bax in the treated cells. The p53 level was significantly resurrected in the mice treated with subtilosome-based siRNA. The siRNA egg-PC-liposome-based formulation also induced the expression of p53 in the treated group. The free form (the naked siRNA-based formulation), did not induce p53 expression in the treated animals. The vehicle controls and untreated groups failed to induce the expression of p53 (wild type) in the animals (Figure 7).

### 2.8. Survival Study

**Figure 8:**
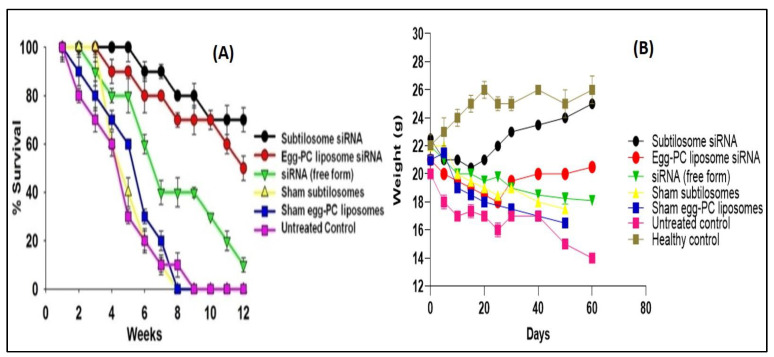
(**A**) Survival graph of the animals belonging to various experimental groups treated with different COX-2-siRNA-encapsulated liposomal formulations. Survival was monitored twice a day for 12 weeks. Each group had 10 animals. Data represented here are the means of three different experiments ± SD (subtilosome siRNA versus free siRNA *p* < 0.005, PC-liposome siRNA versus free siRNA *p* < 0.05, subtilosome siRNA versus PC-liposome siRNA *p* value not significant). (**B**) Animal-weight total. The analysis was conducted every week.

The efficacy of the siRNA-based subtilosome formulation was monitored based on the survival of the HCC-inflicted animals for a period of 12 weeks after the administration of the first dose. The subtilosome-entrapped COX-2-siRNA treated animals had the highest survival rate (80%) followed by the animals treated with the egg-PC-liposome-encapsulated siRNA (50%) (subtilosome siRNA vs. egg-PC-liposome siRNA; *p* value < 0.05). The animals treated with the naked siRNA had a 20% survival rate only (subtilosome siRNA vs. naked siRNA; *p* value < 0.01; Figure 8A). The sham-subtilosome- and -liposome-treated animals did not survive beyond week 9 post-treatment (subtilosome siRNA vs. sham subtilosome; *p* value < 0.001). When exposed to the carcinogenic agent, DEN, the control animal groups (sham-subtilosomes, mixed siRNA, and untreated control) showed a gradual loss in body weight (15–16 g) at day 50 post-exposure. The animals treated with subtilosome-based siRNA mitigated the weight loss to 100% at day 50 post-exposure to DEN (subtilosome siRNA vs. naked siRNA; *p* value < 0.01). The mice treated with siRNA-bearing PC liposomes also regained weight, but to a lesser extent than the mice treated with the subtilosome-based siRNA formulation (subtilosome siRNA vs. scrambled siRNA; *p* value < 0.001; Figure 8B).

## 3. Discussion

Accumulating evidences suggested a challenging role of the cyclooxygenase-2 (COX-2) prostanoid pathway in inflammation and the physiopathology of liver diseases, such as cirrhosis and HCC [12]. The COX-2, an immediate—early-inducible gene, has been reported to be expressed in response to various factors, including mitogens, cytokines, and growth factors [29]. Amongst three different COX isozymes, COX-2 facilitates tumor growth by multiple mechanisms, including angiogenesis stimulation, apoptosis evasion, and the tendency to metastatic and invasive behavior. Considering the indispensable role of COX-2 in cancer pathogenesis, it is tempting to assume that potent inhibitors of this crucial factor may constrain or check cancer-induction processes through various pathways [30,31]. A range of selectively designed COX-2 inhibitors have shown the potential to regulate HCC-cell growth and invasion in animal models [30,31]. However, the chemical nature of the inhibitors makes them prone to drug-resistance-related issues. This eventually made them a less preferred choice for the killing of cancer cells in clinical settings.

Recent scientific advancements suggest that RNAi is one of the promising strategies that may be used to prevent cancer development by inhibiting the expression of various key genes [32]. Despite the associated advantages, siRNA therapy has not been translated successfully in clinical practice due to concerns that need an appropriate resolution. In this regard, specific delivery to target cancer tissues and en route stability issues (*cf.* plasma and other tissue fluids) are of paramount importance. These caveats can be overcome to some extent by using siRNA-encapsulated lipid-based nano-particles. The present study’s results suggested that siRNA-laced subtilosome has desirable pharmacodynamic properties and helps to improve the stability of encapsulated siRNA. This might occur due to the shielding properties of the encapsulating bilayer structure and the site-specific cytosolic delivery of therapeutic siRNA [28]. Studies have shown that the entrapment of siRNA in a nano-carrier not only circumvents its poor plasma-membrane permeability but also facilitates its delivery in the cytosol of the target cells, preventing its premature degradation [33,34]. Among various nanoparticle-based formulations, liposome-based vehicles offer advantages in terms of the biodegradability of the core lipids and lack of immunogenicity [35]. Moreover, strategies based on liposomal-delivery systems hold great promise due to their ability to release entrapped therapeutic agents in a sustained manner and their potential to influence cell-cycle checkpoints to ensure the effective killing of cancer cells. We previously exploited various nanoparticle-mediated drug-delivery systems to accomplish better therapeutic potential in killing cancer cells [28,34,35]. We extended the same approach, envisaging the potential of subtilosomes for delivering encapsulated COX-2 siRNA to the target (cancer cells) for the inhibition of liver carcinogenesis. The DLS analysis established that the particle size of the as-synthesized subtilosomes was ~105 nm (Figure 1). The negative zeta-potential findings suggested that the particles withstood the exposure to the biological-fluid milieu upon their administration to the host. We further analyzed the release kinetics of the formulations to assess the availability of the therapeutic siRNA at the target site. We found that the therapeutic siRNA was released slowly and was sustained over an extended period at different pH (Figure 2). The relatively high release of siRNA at acidic pH might have been directly correlated with the lipid composition of *B. subtilis*. The behavior of the subtilosome may be attributed to the fusogenic phospholipids (phosphatidyl-ethanolamine and cardiolipin), which may facilitate the phase transition of the liposome at low pH of the late endosome and the release of encapsulated siRNA in a pH-dependent manner [36]. The preponderance of phosphatidyl-ethanolamine in the *B. subtilis*-plasma-membrane lipids may have induced the bilayer to undergo an H_II_-phase transition at low pH, which in turn, have induced the fusion of the subtilosome bilayer with that of the endosome membrane. This eventually resulted in the delivery of encapsulated siRNA to the cytosol of the target cells. In fact, cytosolic delivery facilitates the escape of encapsulated siRNA from the endocytic/lysosomal degradation pathway (endosomal release). It is also reported that tumors induce acidic pH in their microenvironment [37]; therefore, this condition might facilitate the localized release of the subtilosome-encapsulated Cox2-specific siRNA at the tumor site (Figure 2). In the next phase of the study, we induced hepato-cellular carcinoma using DEN in model animals. We evaluated the TNF-α expression in the treated animals to assess the efficacy of the formulation in treating induced cancer. TNF-α plays a crucial role in initiating and amplifying inflammatory reactions, leading to tissue destruction and recovery from damage. Upon dysregulation, TNF-α mediates a wide variety of diseases, including cancer [38]. Various reports in the literature suggested that the inflammatory process is inherently associated with many cancer types, including HCC [39]. Different cytokines, such as TNF-α, IL-1β, or IL-18, induce TRAIL expression in HCC cell lines (HepG2, Hep3B, Huh7) upon stimulation [40]. The expression of TRAIL on the HCC cell surface may contribute to tumor-cell immune evasion by inducing apoptosis in activated human lymphocytes [39]. The present study showed that subtilosomes were the most successful in inducing apoptosis in liver-cancer cells, followed by egg-PC liposomes (Figure 5 and Figure 6). The percentage-apoptosis rates were 18.53, 10.43, and 7.52 in the subtilosome siRNA, PC-liposome siRNA, and free form of siRNA-treated groups, respectively (Figure 5). The probable explanation for the superior activity observed is their stability, as well as their capacity to deliver encapsulated siRNA at the target site. The naked siRNA did not exhibit significant apoptosis induction, and the sham liposomes did not affect the process of apoptosis induction.

Apart from apoptosis induction, we also elucidated the potential of subtilosome-based siRNA to modulate cell-signalling pathways in the host, ensuring the cell-cycle arrest of the treated cancer cells. Apoptosis and cell-cycle regulation are two delicately poised processes that play a crucial role in inhibiting cancer cells. Cell-cycle checkpoints are generally regulated in healthy cells by the active involvement of chemical mediators and other related factors. This helps to maintain a delicate equilibrium that may aid in cell differentiation, survival, and apoptosis. The p53 pro-apoptotic gene has been suggested to play a crucial role in regulating the cell cycle and induces apoptosis in response to DNA damage [41]. If the normal expression of the functional wild-type p53 (p53wt) gene is inhibited, this leads to the uncontrolled growth of tumor cells. In fact, mutations in the p53 gene or its abnormal functioning may lead to malignancy in the related host [42]. Bax is a known p53 target and becomes trans-activated during p53-mediated apoptosis [43]. The Bax- and bcl-2-family proteins also play a crucial role in apoptosis regulation [44]. In general, bcl-2 and its related regulatory factors suppress apoptosis; on the other hand, the over-expression of the bax gene inhibits bcl-2 function and promotes apoptosis [45]. Among various treated groups of animals, the highest p53wt expression was observed in the subtilosome-siRNA-treated animals, followed by the egg-PC-liposome siRNA (Figure 7).

Naked siRNA delivery could have induced the insignificant expression of p53 in the treated animals (Figure 7). Interestingly, the subtilosome-based siRNA-nanoparticle formulation also successfully induced the elevated expression of bax compared to that of the free form of the siRNA (*p* value < 0.01). The up-regulated expression of the bax in the animals treated with subtilosome-siRNA nanoparticles (Figure 7) suggests the putative involvement of the mitochondrial (intrinsic) pathway in the apoptosis as it ensued in the release of cytochrome c, which in turn can activate related caspases in the treated cells [46]. The COX-2 gene is a downstream target gene of p53. Its expression is induced by the p53-mediated activation of the Ras/Raf/MAPK pathway. It has been demonstrated that COX-2 inhibits DNA damage or p53-induced apoptosis in loss-of-function models of COX-2 [47]. The down-regulation of COX-2 expression demonstrates the superior anti-cancer efficacy of subtilosome-siRNA nanoparticles compared to PC-liposome-siRNA and free siRNA. These results suggest that liposome-mediated COX-2 delivery successfully induces apoptosis in liver-cancer cells. The observed up-regulation of p53wt might be correlated with homeostasis involving the Ras/Raf/MAPK pathways.

Considering the etiology of HCC and its correlation with overall liver function, we determined the level of various associated enzymes in both the siRNA-treated and the control animals. We selected liver enzymes as a parameter to check liver carcinogenesis. We found that the subtilosome-based formulation might deliver COX-2-specific siRNA much more efficiently than the treatment with free siRNA and helped in the regression of the liver enzymes (ALT and AST) in the HCC animals (Figure 4). The sham PC liposomes and sham subtilosomes had no effect on the liver enzymes and behaved as an untreated group; this might be attributed to the sheath effect of the liposomes that successfully delivered encapsulated siRNA to the tumor site. Being more stable, the subtilosome group showed more enzyme reduction than that of the egg-PC liposomes. The siRNA-bearing subtilosome formulation was found to be the most successful in normalizing the levels of liver enzymes. The free form of the siRNA could not normalize the enzyme levels as it failed to reach target cancer cells, partly due to its destruction by serum endonucleases.

Among the various liposome-based siRNA formulations, the efficacy (in terms of survival rate) of the subtilosome-encapsulated COX-2 siRNA was found to be superior to the PC-liposome-entrapped siRNA and free siRNA (Figure 8). In concordance with our earlier study [28], we observed that the animals treated with COX-2 siRNA-bearing subtilosomes possess an 80% survival rate. By contrast, those treated with the liposome-entrapped siRNA and free siRNA exhibited only 50% and 20% survival rates, respectively. We also studied the weights of the vital organs of the treated animals to assess the toxic effects of our formulations. We found insignificant changes in the importance of the kidneys and spleen compared to those of the normal group, demonstrating that none of these treatments affected the weights of the organs (Figure 8).

The potentially higher anti-cancer efficacy of subtilosme-siRNA nanoparticles might be attributed to their better fusogenic character, which enables greater siRNA delivery to tumor tissues. The fusogenic property of subtilosome was determined by the FRET, dequenching assay, and content-mixing assay (Figure 3). In brief, the lipid composition of liposomes plays a detrimental role in determining their fusogenicity. *B*. *subtilis* has PE as the main lipid component of its membrane. The phospholipid, PE, is thought to play a significant role in membrane–membrane fusion [48] by inducing negative membrane curvature [49], which helps in the attainment of the stalk-like state that may act as an early intermediate during the process of fusion [50,51]. During the union, PE interacts with surrounding water molecules less firmly in a more energetically favorable condition due to its peculiar shape and charge. Hence, it circumvents the repulsive hydration forces ensuing in the fusion of the approaching bilayers [50]. Further, the PE phospholipid in the inverted hexagonal (HII) phase enables the formation of membrane defects in the bilayer, thereby bringing the bilayers of two fusing vesicles into close proximity [52]. The ratio of head groups measured between 0.9 and 2.045 among two phospholipids, namely phosphatidyl-choline (PC) and PE, play a crucial role in membrane–membrane fusion (synaptic vesicles, synaptic membranes, and viral membranes).

The *B*. *subtilis* contains at least five major phospholipids, of which PE and PG form the bulk of the membrane [53]. These phospholipids are present in other bacilli, such as *B. megaterium* [54], *B. licheniformis* [55], and *B. cereus* [56]. Interestingly, we and others previously demonstrated that liposomes made up of lipids of *E. coli* (escheriosomes) and archaebacteria lipids (archaeosomes) can efficiently deliver their contents [57,58]. Since *B. subtilis* and *E. coli* share similar lipid profiles, with PE as the predominant lipid [59], we hypothesized that liposomes that are composed of the total polar lipids of *B. subtilis* (subtilosomes) might have similar potential as escheriosomes. We have previously showed that subtilosomes undergo spontaneous membrane fusion with macrophages, resulting in the delivery of their entrapped material into the cytoplasmic compartments of target cells [23].

It can be speculated that the targeted delivery of subtilosomes can be further enhanced by grafting cancer-cell-surface-specific antibodies/aptamers or precise ligands specific to certain receptors present on cancer cells. Recent interest has focussed on exploring the possibility of increasing siRNA efficacy by several folds compared to that of the free form of siRNA. Finally, we can conclude that since subtilosomes are more stable and have the potential to undergo efficient membrane–membrane fusion, they may deliver COX-2 siRNA to the cytosol of the target cancer cells much more effectively than egg-PC liposomes, thereby helping in cancer regression, with increases in the survival of the treated animals.

## 4. Materials and Methods

All the reagents used in the study were of the highest purity available. The siRNAstargeting COX-2 gene was designed by and purchased from Santa Cruz Company (Finnell Street Dallas, TX, USA). The COX-2 siRNA target sequence was 5′-GCTGGGAAGCCTTCTCTAA-3′, the sense strand was 5′-GCUGGGAAGCCUUCUCUAAdTdT-3′, and the antisense strand was 5′-dTdTCGACCCUUCGGAAGAGAUU-3′. Anti-p53 wild type (wt), anti-Bax, anti-Cox-2, and anti-β-actin antibodies were purchased from BD Biosciences (San Diego, CA, USA). Liver enzymes were estimated using the kits from Span diagnostics (Bengaluru, India). L-Aminonapthaline-3,6,8-trisulfonic acid (ANTS) and *N*,*N*′-p-xylylene bis (pyridinium bromide) (DPX) were bought from Molecular Probes, Inc. The fluorescent probes, Rh-PE and NBD-PE (Avanti polar lipids), were kind gifts from Dr. Anu Puri (NIH, Frederick, MD, USA). All other reagents were of analytical grade and purchased from local suppliers.

### 4.1. Animals

The BALB/c male mice weighing 20 ± 2 g were obtained from the animal-house facility of the IBU, AMU. The animals were housed in polypropylene cages on wood-powder bedding under an air-conditioned ambiance. Animals were quarantined in equal light/dark cycles (12/12 h) and had free access to a standard dry-pellet diet (Ashirwad, Chandigarh, India) and water ad libitum. Animals were examined for mortality and morbidity before the commencement of the study, and only healthy animals were included in the experiments. The animals were anaesthetized with 5 mg/kg ketamine and 4 mg/kg xylazine before being euthanized. Humane endpoints were applied for mice that survived at the end of the experiment. In all the experimental procedures, efforts were made to minimize animal pain and suffering. All animal experiments were reviewed and approved by the Institutional Animal Ethics Committee of the Interdisciplinary Biotechnology Unit, Aligarh Muslim University, India. The techniques used to administer various formulations to animals were strictly performed according to the national regulatory guidelines issued by the Committee for Control and Supervision of Experiments on Animals (CPCSEA), Government of India (approval identifier 332/CPCSEA).

### 4.2. Isolation of B. subtilis Membrane Lipids and Preparation of siRNA-Bearing Subtilosome Formulation

The *B. subtilis* was cultured in nutrient broth (1% peptone, 0.3% beef extract, 0.3% yeast extract and 1% sodium chloride, pH 7.4). Phospholipids were isolated using the method of Bligh and Dyer [60], modified in our laboratory. Briefly, the cells were harvested from the mid-log phase of a batch culture and pelleted at 2000× *g* for 10 min. The cell pellets were washed with normal saline (NS), suspended in methanol, and ruptured for 60 min in a bath-type sonicator (Heat System-Ultrasonics W-385, Oregon, USA) at 4 °C. The cell debris was removed through filtration, and the filtrate was washed with 0.2 times the volume of the filtrate of NS. The chloroform-soluble lipids were dried using a rotatory-evaporator (Büchi, Switzerland) and kept at −20 °C until further use. The lipids were analyzed through two-dimensional thin-layer chromatography (2-D TLC). The lipids (5 mg) were applied to a 20 × 20-cm, 0.25-mm silica-gel 60-glass plate (Merck, NJ, USA). The plate was developed with chloroform–methanol–ammonia (65:35:5) in the first direction and then with chloroform–methanol–water (60:30:4) in the second direction. The lipids were visualized with phosphomolybdic acid-spray reagent (Sigma, NJ, USA). Phosphatidylethanolamine (PE), phosphatidylglycerol (PG), and cardiolipin (CL) were used as standard references. All the standard references were purchased from Sigma. The phospholipid composition was determined by the method of Kumar and Gupta [61].

The lipid solution was reduced to a thin, dry film and hydrated with NS, followed by sonication under a nitrogen atmosphere to obtain plain subtilosomes. The empty subtilosome formulation was mixed with an equal volume of siRNA solution (10 mg/mL). The mixture was flash-frozen and thawed three times and then lyophilized. The free-flowing dried powder, obtained after lyophilization, was rehydrated with distilled water (original volume) and passed through a polycarbonate membrane filter to obtain a homogenous formulation. The final preparation was centrifuged at 14,000× *g*, and the pellet was further washed at least three times with NS to remove the traces of non-trapped siRNA.

### 4.3. Preparation of Egg-PC-Liposome-Encapsulated siRNA

The siRNA-entrapped egg-PC liposomes were prepared from egg PC (49 µmoL) and cholesterol (21 µmoL) using published methods standardized in our laboratory [28,62]. Briefly, lipids (20 mg) were dissolved in a minimum volume of chloroform: methanol (1:1 *v*/*v*). The solvent was evaporated carefully under reduced pressure to form a thin lipid film on a round-bottomed flask wall. Finally, traces of organic solvents were removed, subjecting the flask to a high vacuum at 4 °C overnight. Subsequently, the dried lipid film was hydrated with 2.0 mL of 150 mM sterile NS with intermittent vigorous agitation, followed by sonication for 30 min at 4 °C in a bath sonicator under a nitrogen atmosphere. The sonicated preparation was centrifuged at 10,000× *g* for 30 min at 4 °C to remove non-dispersed lipids. Next, the siRNA solution in NS was mixed with PC liposomes. The mixture was frozen and thawed several times and finally lyophilized to obtain a free-flowing dry powder. The powder was reconstituted with distilled water (1:10 of the original solution) to obtain the egg-PC-liposome-based siRNA formulation. This was used as a positive control.

### 4.4. Characterization of the siRNA-Bearing Formulations

The zeta potential of the subtilosome-based siRNA nanoparticles was measured with the Zetasizer Nano ZS instrument (Malvern Instrument Limited, Worcestershire, UK). Electron microscopy of the subtilosome-siRNA nanoparticles was conducted to characterize the nanoparticles’ size, surface morphology, and shape. For DLS, the lyophilized preparation of the subtilosome-siRNA nanoparticles was suspended in distilled water (2 mg/mL), and a single drop was analyzed with the particle-size analyzer, NANOPHOX (Sympatec GmbH, Clausthal-Zefferfeld, Germany).

### 4.5. Entrapment Efficiency of siRNA

The entrapment of siRNA in various liposome preparations was assessed with a published protocol standardized in our laboratory [28]. Briefly, an aliquot of liposomes was dissolved in 0.1% Triton X-100 followed by an analysis of the siRNA content through absorbance determination with a UV spectrophotometer (Shimadzu, Tokyo, Japan). The entrapment efficiency (% EE) was calculated as the percentage ratio between the amount of entrapped siRNA and the total amount of the siRNA.

### 4.6. In Vitro Release Kinetics

To assess the release kinetics of siRNA from subtilosomes at various pH, multiple weighed aliquots of the nanoparticles were dispensed in separate micro vials. A volume of 1.0 mL of 10 mM sterile normal saline (pH 7.0) was added to each vial, which was further placed in a dialysis bag fully immersed in release medium (NS, pH 7.0) under gentle stirring on a magnetic stirrer at 37 °C. Aliquots of the release medium were withdrawn for analysis at various times and replaced with a fresh medium. The experiment was conducted for 72 h, and the absorbance of the collected samples was determined at 260 nm. The calculated amount of the released siRNA was plotted against time.

Similarly, the release kinetics were examined in 0.1 M HEPES buffer (pH 5), and serum. All the determinations were performed in triplicates. Results are reported as the mean ± SD.

### 4.7. High-Performance Liquid Chromatography (HPLC) Analysis

Purification of siRNA was assessed by performing high-performance liquid chrom- atography (HPLC) analysis as per the standardized protocol of our lab. The HPLC was performed employing MicroBondapak C-18 column (30 cm ×4 mm (diameter); particle size 5 µm) procured from Waters Associates, Milford, MA, USA. The mobile phase A was 0.1 M triethylamine acetate (TEAA) with pHof 7.5 and mobile phase B was 20% acetonitrile added to A. The column temperature was maintained at around 60 °C. The elution volume was delivered at a rate of 1 mL/min. The retention time of siRNA was about 8 min. By comparing the siRNA peak at 260 nm with a standard of known concentration eluted at the same retention duration, the results were quantified.

### 4.8. Determination of Fusogenic Property of Subtilosomes

#### 4.8.1. Fluorescence-Resonance Energy Transfer (FRET) Assay

The lipid mixing between the NBD/rhodamine-labeled subtilosomes (<600 nmoles, lipid P) and unlabeled subtilosomes was followed by monitoring FRET between NBD (absorption, 470 nm; emission, 520 nm) and rhodamine (absorption, 536 nm; emission, 585 nm). The FRET was measured by mixing labeled subtilosomes with unlabeled subtilosomes in molar ratios of 1:10, 1:4, 1:2, and 1:1. The excitation wavelength was chosen to be 20 nm below the absorption maxima of NBD to allow a better resolution between the scattered light peak and the NBD emission peak, as well as to minimize the direct excitation of rhodamine. Finally, the fluorescence was monitored at 520 nm. The efficiency (E) of the FRET was calculated as follows:

*E* = 1 − *F/F_t_*

where ‘*F*’ is the NBD fluorescence in the presence of rhodamine and ‘*F_t_*’ is the NBD fluorescence at maximal dequenching, which was measured after disrupting vesicles with Triton X-100 (1% final concentration).

#### 4.8.2. Dequenching Assay

Vesicle–vesicle fusion was determined by including self-quenching concentration (5 mol%) of Rh-PE in subtilosomes or egg-PC liposomes. The fluorescence dequenching was measured by mixing labeled subtilosomes or egg-PC liposomes with their unlabeled counterparts in a molar ratio of 1:10. The fluorescence associated with the labeled subtilosomes or egg-PC liposomes was monitored for up to 20 min using excitation and emission wavelengths of 536 and 585 nm, respectively, and percentage dequenching was calculated as follows:

*% dequenching* = 100 × (*F* − *F*_0_
*)/(Ft − F*_0_
*)*

where *F*, *F*_0_, and *F_t_* are the fluorescence intensities at time ‘*t*’ and 0 min, and after disrupting the vesicles with Triton X-100 (1% final concentration), respectively.

#### 4.8.3. Aqueous-Content-Mixing Assay

Quenching of the ANTS fluorescence by DPX was monitored in order to follow the mixing of the aqueous contents of the vesicles undergoing fusion. The ANTS-containing subtilosomes or egg-PC liposomes were mixed with a 10-fold excess of the DPX-containing subtilosomes or egg-PC liposomes in a total volume of 3 mL. The mixture was incubated at 37 °C and the ANTS fluorescence was measured at varying periods of time. The ANTS fluorescence observed at zero minutes was taken as 100% fluorescence, while the fluorescence values observed after lysing a mixture of ANTS-containing and DPX-containing with Triton X-100 (1% final concentration) were taken as 0% fluorescence. The excitation and emission wavelengths used were 380 nm and 540 nm, respectively.

### 4.9. Induction of Liver Cancer with Diethylnitrosamine (DEN)

Liver cancer was induced in animals following a published protocol standardized in our laboratory [63]. Briefly, a single dose of DEN (120 mg/kg) was injected through the intraperitoneal route. After 40 days of incubation, the animals were given the treatment with various formulations. Liver-cancer induction was assessed through the determination of liver enzymes and tissue histology.

### 4.10. Assessment of Anticancer Efficacy

The efficacy of the siRNA-based liposomal formulations was assessed by examining various parameters, including liver enzymes, percentage of survival, liver histology, and western blot profile of Cox-2 and various apoptosis-related molecules. The siRNA was used at a dose of 60 mg/kg of body weights of mice. All the siRNA formulations were given through the intravenous route (i.v.) for 10 days after induction of the tumor and then were left for a resting period of 15 days to observe the effect of the formulations. For the study, BALB/c mice were divided into 7 groups of 10 animals each, as follows:

Group I Healthy control;
Group II Untreated control (DEN-treated only);
Group III Sham liposomes;
Group IV Sham subtilosomes;
Group V siRNA (free form);
Group VI PC-liposome siRNA; and
Group VII Subtilosome-siRNA

### 4.11. Assessment of Liver Enzymes

As liver enzymes are up-regulated in cancer, the level of various enzymes was determined to follow the progress of the disease [64]. The efficacy of the siRNA-based nanoparticle formulations was assessed 15 days after the last treatment by monitoring the levels of various liver enzymes, namely, aspartate transaminase (AST) and alanine transaminase (ALT), in treated BALB/c strain of mice. All analyses were carried out in triplicates (*n* = 3).

### 4.12. Measurement of TNF-α Level

The TNF-α concentration was measured in serum samples of animals from various treatment groups using ELISA (BD Biosciences) [65]. The ELISA was performed as per the manufacturer’s guidelines. Briefly, 50 μL of the purified capture antibody was coated on polystyrene microtiter plates at 4 °C in carbonate buffer pH 9.4 overnight. After incubation, the plates were washed five times with PBST and blocked with 5% skimmed milk. After 1 h of incubation at 37 °C, the plates were washed with PBST and incubated with 50 μL of the sample for the detection of cytokines. After 2 h of incubation at 37 °C, the plates were washed and coated with biotinylated anti-mouse TNF- α (detection antibody). The plates were washed thrice with PBST, and 100 μL of streptavidin-HRP were added to each well. The plates were incubated for 30 min at room temperature, followed by three rinses with PBST, and finally developed with tetra-methyl-benzidine. The absorbance was determined at 450 nm using a microtiter plate reader. Known concentrations of TNF-α were used as standard.

### 4.13. Preparation of Cell Homogenate

The livers were removed from experimental mice and transferred to various sample tubes on ice. The samples were homogenized, and the nuclear fraction was prepared using RIPA buffer. The protein content of the homogenate was determined using bovine serum albumin as a standard [66].

### 4.14. Western Blotting

The cell homogenate was analyzed for various apoptotic molecules using Western blotting [67]. Briefly, proteins (30 µg/well) were resolved on 10% SDS-PAGE under non-denaturing conditions and electroblotted onto nitrocellulose membranes. The blots were blocked overnight with 5% non-fat dry milk and probed with appropriate antibodies at the dilutions recommended by the suppliers. To quantify equal loading, membranes were probed with the antiα-tubulin antibody. The intensity of the band was quantified using image-analysis software from the Image Gel Documentation System. Data are presented as the relative pixel density of each band normalized to a band of α-tubulin.

### 4.15. Apoptosis Detection with APO-BRDU^TM^ Labeling

Liver cells were isolated after 15 days of the resting period using the protocol described and modified in our laboratory [68]. Apoptosis in cancer cells from various formulation-treated groups was analyzed using the APO-BrdU^TM^ kit (BD Biosciences). Briefly, cancer cells (1 × 10^6^ cells/mL) were suspended and fixed using 1% paraformaldehyde (dissolved in PBS pH 7.4). Suspended cells were placed on ice for 30–60 min. After fixation, cells were centrifuged at 500× *g* for 5 min, and the supernatant was discarded. Cells were washed thrice with PBS, resuspended in 70% (*v*/*v*) ice-cold ethanol, and incubated in ice for 30 min. Cells were then centrifuged at 300× *g* for 5 min and washed three times after removing the ethanol. Subsequently, the cell pellet was incubated with 50 µL of DNA labeling solution at 37 °C for 60 min. At the end of the incubation period, 1.0 mL of rinse buffer was added, and tubes were centrifuged at 300× *g* for 5 min. This exact process was repeated three times, and finally, the cell pellet was incubated with 0.1 mL of FITC-labeled anti-BrdU antibody in the dark for 30 min at room temperature. At this point, cells were further analyzed with flow cytometry.

### 4.16. Survival Analysis

The survival of animals treated with various siRNA-bearing subtilosome-based formulations was considered a measure of its efficacy. The animals were monitored twice a day for their mortality for 12 weeks after the beginning of the therapy. The weights of the individual mice were monitored for 8 weeks.

### 4.17. Statistical Analysis

One-way ANOVA was used for comparing the mean values of tumor volume between various groups after assessing the homogeneity of the variance between treatments. Post hoc analysis comparing the two groups was conducted using the least statistical difference (LSD) technique. The Kaplan–Meier analysis was used to estimate the survival of tumor-free animals, and differences were analyzed with the log-rank test.

## Data Availability

The data in this study can be availed by request from the corresponding author.

## Abbreviations

ALT: Alanine transaminase; ANTS: L-Aminonapthaline-3,6,8-trisulfonic acid; AST: Aspartate transaminase; CL: Cardiolipin; COX-2: Cyclo-oxygenase-2; DEN: Diethylnitrosamine; DPX: *N*,*N*′-p-xylylenebis (pyridinium bromide); HCC: Hepatocellular carcinoma; Rh-PE: *N*-(lissamine rhodamine B sulfonyl) phosphatidylethanolamine; NBD-PE: L-(phophatidyl ethanolamine-*N*-(4-nitrobenzo-2-oxa-1,3-diazole); NS: Normal saline; PE: Phosphatidylethanolamine; PG: Phosphatidylglycerol; siRNA: Small interfering RNA.
